# “You Need ID to Get ID”: A Scoping Review of Personal Identification as a Barrier to and Facilitator of the Social Determinants of Health in North America

**DOI:** 10.3390/ijerph17124227

**Published:** 2020-06-13

**Authors:** Chris Sanders, Kristin Burnett, Steven Lam, Mehdia Hassan, Kelly Skinner

**Affiliations:** 1 Department of Sociology, Lakehead University, 955 Oliver Road, Thunder Bay, ON P7B 5E1, Canada; 2 Indigenous Studies, Lakehead University, 955 Oliver Road, Thunder Bay, ON P7B 5E1, Canada; kburnett@lakeheadu.ca; 3 Department of Population Medicine, University of Guelph, 50 Stone Road E., Guelph, ON N1G 2W1, Canada; lams@uoguelph.ca; 4 Social Justice Studies, Lakehead University, 955 Oliver Road, Thunder Bay, ON P7B 5E1, Canada; mhassan6@lakeheadu.ca; 5 School of Public Health and Health Systems, University of Waterloo, 200 University Avenue West, Waterloo, ON N2L 3G1, Canada; kskinner@uwaterloo.ca

**Keywords:** North America, personal identification, rural, scoping review, social determinants, urban

## Abstract

Personal identification (PID) is an important, if often overlooked, barrier to accessing the social determinants of health for many marginalized people in society. A scoping review was undertaken to explore the range of research addressing the role of PID in the social determinants of health in North America, barriers to acquiring and maintaining PID, and to identify gaps in the existing research. A systematic search of academic and gray literature was performed, and a thematic analysis of the included studies (*n* = 31) was conducted. The themes identified were: (1) gaining and retaining identification, (2) access to health and social services, and (3) facilitating identification programs. The findings suggest a paucity of research on PID services and the role of PID in the social determinants of health. We contend that research is urgently required to build a more robust understanding of existing PID service models, particularly in rural contexts, as well as on barriers to accessing and maintaining PID, especially among the most marginalized groups in society.

## 1. Introduction

Personal identification (PID) serves multiple and frequently contradictory purposes within the context of the modern state. Establishing the identity of individuals by connecting them to key information such as age, sex/gender, birthdate, nationality, and residence, PID has the potential to confer certain rights and privileges on individuals. Common forms of PID, like birth certificates, passports, driver licenses, and government-issued health cards, grant access to important benefits, such as health and social services, while non-government PID (e.g., private club membership card, credit cards) typically allow situational access or benefits to the bearer. However, the loss and misuse of personal identity can have devasting consequences for individuals.

State record keeping also makes individuals visible to the polity and, thus, governable and subject to the loss of freedoms. Recent conversations about the significance of PID and identification security processes, especially since the events of 9/11 and the ongoing collection and sale of personal data through large online social media platforms, have largely centered on fears about increased state and corporate surveillance, as well as identity theft and fraud [1,2,3]. While it is extremely important to acknowledge that these circumstances have had and continue to have enormous and detrimental impacts on the lives and well-being of individuals and communities, largely overlooked in these conversations is the central role played by PID in accessing essential state services, particularly among people who are socially and economically disenfranchised. Taylor and colleagues have astutely described this phenomenon as the inherent ambiguity of viewing citizens as both a “risk to be managed, and thereby an object for surveillance (and) a consumer deserving the best possible public service” [4] (p. 152). After all, without PID it is nearly impossible to access health care, housing, income maintenance, education, banking services, employment, and pension benefits, among other essential programs and services. It is also frequently impossible to access something as simple as emergency food services, like a food bank, without providing government-issued PID and proof of residence.

While access to and possession of PID does not of itself guarantee education, health, protection, and participation in society for marginalized people, not having certain forms of government-issued PID ensures that access to essential health, social, and financial services is nearly impossible [5]. Possession of PID, in effect, becomes the gateway to accessing the social determinants of health, particularly in rural settings [6]. Thus, what we refer to as the “problem of personal identification” occurs when populations that are already marginalized and underserved are made further vulnerable because they lack forms of official identification that enable them to secure vital benefits and resources, effectively making them invisible to health and social services.

Literature on PID in the North American context that does not examine this issue from a security/governmentality perspective tends to focus on populations that are precariously housed or homeless and living in urban spaces. Barriers to PID associated with people living in rural and northern settings in North America have not been adequately explored. Additionally, relatively little attention is paid to the particular PID challenges experienced by people who are racialized and Indigenous, and further, how those identities operate alongside space and gender. These multiple and intersecting identities are important to explore with regard to PID because, as Audre Lorde points out, “we do not live single-issue lives,” [7] meaning that it behooves us to understand the dynamic ways that lived identities and structural systems intersect to the detriment of the most marginalized individuals and social groups.

Through this scoping review, we seek to enter into this conversation regarding barriers to obtaining PID by highlighting the ways in which the problems posed by a lack of PID are particularly pronounced for people living in rural, northern, and remote access communities—people whom we already know experience poorer health outcomes than residents in metropolitan and suburban areas, and whom to date have been largely ignored in the scholarship [8]. Further, in Canada, Indigenous people are more likely to reside in the provincial north and territories than non-Indigenous people [9,10,11]. Given the higher proportion of Indigenous people and communities located in rural and remote areas, we contend that health disparities and a lack of access to health and social services resulting from a lack of PID exacerbate inequalities between Indigenous and non-Indigenous people, broadly speaking.

A better understanding of the problem of PID is needed, particularly as it pertains to accessing health and social services for the most marginalized people and groups in society. The aims of this scoping review are as follows: First, to provide readers with a clear understanding of the current research on this topic by providing a comprehensive review and analysis of the academic and gray literatures on the barriers to attaining PID in North America. Second, this review aims to show the significance that a lack of PID has for people’s ability to access health and social services. Third, this review aims to identify gaps in the existing research, particularly in regards to rural and Indigenous peoples and communities. Fourth, we discuss the implications for rural and Indigenous communities and identify future directions for research on PID.

## 2. Materials and Methods

Scoping reviews aim to provide a survey of studies on or related to a topic rather than to assess the quality of each study. A scoping review was considered an appropriate strategy for this research topic because it was not previously comprehensively reviewed [12]. This scoping review was conducted following guidelines for scoping studies outlined by Colquhoun and colleagues involving a stepwise process of search, selection, extraction, and synthesis of the literature [13]. A separate protocol for this review does not exist; below, we provide a detailed roster overview of the review process. To ensure the quality of scoping review reporting, we used a checklist developed by Tricco and colleagues [14]. Other than the assessment of and secondary analysis of the studies, this scoping review complies with the Preferred Reporting Items for Systematic Reviews and Meta-Analyses (PRISMA) statement and checklist.

### 2.1. Definitions of Key Terms

We use the term “PID” to refer to all types of government-issued personal identification used to recognize citizens and denizens for the purposes of granting access to vital services. Common forms of PID include “identity documents” (e.g., birth certificates, passports) and “identity cards” (e.g., driver licenses, provincial health cards, hunting licenses). By contrast, the term “ID” includes non-government issued forms of identification (e.g., student body card, private club membership, employment ID cards) and may not grant access to vital services provided by the government. We only use the term “ID” when quoting studies or in reference to specific identification cards. Because existing studies sometimes use these terms interchangeably, or simply use the more common term ID, our search strategy employed the term “identification” to search databases.

### 2.2. Search Strategy

A search string was developed (Table 1) and used to search the following citation databases: Web of Science™ (Clarivate Analytics, Philadelphia, PA, USA), CORE Collection (EBSCO, Ipswich, MA, USA), MEDLINE^®^ (National Library of Medicine, Bethesda, MD, USA), CabDirect© (CABI, Wallingford, UK), and EBSCOHost© (EBSCO, Ipswich, MA, USA). These databases cover health, sociology, anthropology, and psychology disciplines, thereby providing the opportunity to capture the broad literature, as well as approach the research question from different perspectives. No search restrictions were placed (e.g., language, date, publication type). A complementary search for gray literature documents, such as government research reports, was also conducted using a series of simple search strings in Google (e.g., “barriers to obtaining identification in North America”). As Google returns results based on relevance criteria related to the search term entered, only the first 100 hits of each search were examined [15]. The reference list of all relevant studies was also hand-searched to identify any further relevant studies not captured in the search. Records were uploaded into EndNote X7^®^ and de-duplicated.

### 2.3. Relevance Screening

The titles and abstracts of studies were screened according to a priori inclusion criteria. To be included in the scoping review, studies needed to report on barriers or facilitators to obtaining PID in the North American context. Studies were excluded if they were not relevant to this topic or were not in the English language. In some cases, a full-text review was conducted in order to assess suitability. Sources of evidence included primary studies published in English as journal articles, books, research reports, dissertations and theses, or conference proceedings. To ensure the availability of data for charting purposes, we excluded newsletters, news articles, and summaries.

### 2.4. Data Charting

We developed and used a charting form to capture data from each study. Key information extracted included author, year of publication, country of origin, purpose, publication type, study scale, study population, methodology/methods, and key findings that related to the scoping review question. Charting followed an iterative process in which the data were extracted and the charting form was updated continuously. Of note, study screening and data charting were done by one author (S.L.), presenting possible concerns over reviewer bias. To address this bias, this author discussed challenges and uncertainties related to the reviewing strategy with the co-authors and refined the approach in the process.

### 2.5. Analysis

The data analysis included quantitative analysis and qualitative analysis. For quantitative analysis, we used descriptive statistics to present the characteristics of the study, methodology, and findings. To characterize and summarize factors which act as barriers to and facilitators of obtaining identification, we used thematic qualitative analysis following a process outlined by Braun and colleagues [16]. First, studies were read in full and notes were written to facilitate data familiarization. Then, codes were assigned to portions of the text that discussed identification. We used an inductive approach to coding, with no pre-formulated assumptions of how codes should be defined. Similar codes were then grouped into descriptive themes that illuminate patterns in the data across studies. We selected quotations that exemplified these themes and presented them in the results to provide a rich and nuanced description of the data [16]. To ensure the validity of the qualitative analysis, we held regular discussions among the authors surrounding the developed themes. Data were stored in a spreadsheet (Excel 2013, Microsoft Corporation, Redmond, WA, USA) to facilitate analysis.

## 3. Results

**Figure 1:**
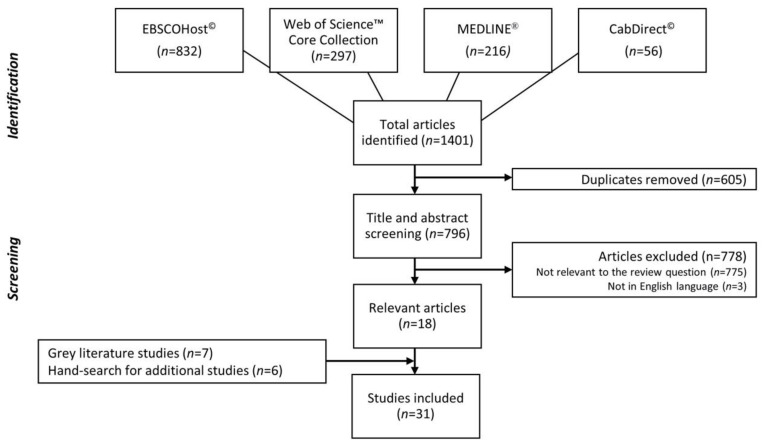
Flow chart of the selection of studies on barriers to and facilitators of obtaining identification in North America.

The initial search returned 1401 studies; after the removal of duplicates and non-relevant studies, a total of 31 studies were included (Figure 1). A summary of the descriptive characteristics of these studies is shown in Table 2. The median publication year of relevant studies was 2005 (range 2000–2018). There was a near equal balance of publications from Canada (51%) and the United States (49%). Most of the studies were from the academic literature, though a significant portion (30%) were from the gray literature. Many of the 31 studies (45%) were purely qualitative and used interviews to collect qualitative data from participants. Most studies focused on homeless youth, adults, or people in general (58%, *n* = 18). A detailed summary of the 31 studies, including relevant findings, can be found in Appendix A.

Three descriptive themes were identified across the 31 relevant studies that capture barriers to, and facilitators for, obtaining identification: (1) gaining and retaining identification, (2) access to health and social services, and (3) facilitating identification programs and services. These themes are described in detail below and are supported by illustrative quotations from study participants and/or study authors.

### 3.1. Gaining and Retaining Identification

One of the biggest challenges identified in the literature that individuals faced was the acquisition and retention of PID. According to many studies (32%, *n* = 10), the main reason people reported for not having identification was that it had been either lost or stolen (e.g., [17,18,19]). This is particularly true for many people who are precariously housed or homeless. Campbell and colleagues, for example, conducted one-on-one interviews and focus groups with individuals in Calgary that were homeless and health and social services providers in which one participant without housing identified PID as a key barrier: “One of the things I just thought of that could be a potential barrier is missing or stolen ID” [17] (p. 7). Further support is provided by a survey of 1169 people who were homeless in Toronto, which found that 315 (27%) were not in possession of their health card [18], and in the United States, an estimated 11% of voting-age citizens lacked identification, with estimates higher among those experiencing homelessness [20]. Additionally, it is common in homeless shelters to have one’s personal belongings, where IDs and other personal documents are typically stored, taken if left unattended for even a short period of time or while sleeping [21,22,23]. Consequently, whether living on the streets or staying in a shelter, maintaining possession of one’s belongings requires constant vigilance, which is challenging for many people living in precarious circumstances. In addition, many people experiencing homelessness do not possess the means of replacing their PID (e.g., money for fees, knowledge of application process, competency with bureaucratic forms).

Other studies (16%, *n* = 5) highlighted the requirement of an address or an existing piece of identification in order to apply for additional identification (e.g., [24,25,26]); yet, many homeless people frequently are unable to provide either of these. Gordon interviewed 102 people visiting identification clinics in Edmonton, Alberta, and reflected: “Nine people spontaneously told me ‘you need ID to get ID,’ or similar words” [27] (p. 256). In a study exploring the lived experiences of 20 adolescent women in Seattle who were homeless, the authors reported:

*[The young women] claimed that the biggest structural barriers to care [that they identified] at many hospitals or clinics not designed for homeless youth were questions over consent for care, being asked to provide addresses and an identification (ID) card, and source of insurance or payment [28] (p. 169).*

Still more studies (16%, *n* = 5) emphasized the high cost of obtaining identification (e.g., [25,26,29]). For example, one study from Toronto, Canada finds:

*Even a modest fee can make it difficult for a homeless young person to obtain identification—and in many states, the cost of obtaining an ID card is far from modest [25] (p. 18).*

For people who are economically marginalized and/or precariously housed, even seemingly minor fees constitute a financial hardship that makes the acquisition of PID prohibitive. In the province of Ontario, for example, higher fees are charged for replacement birth certificates, and if people go through “third party” providers rather than state agencies to obtain this form of ID, additional service fees are incurred. This means that people who have little or no money and who are likely to lose or have their PID stolen due to being precariously housed are further burdened with higher replacement fees. Ultimately, people regularly prioritize the immediate needs of food, transportation, or rent rather than the costs of replacing a lost or stolen document. Furthermore, additional costs are required if individuals must take public transportation or live in rural or remote locations and have to travel to service centers. According to a United Nations report, the “greater the distance to the registration center the higher the financial costs to the family” [30].

Other scholarship outlined those barriers to obtaining identification that were unique to specific social groups. For example, a lack of legal identity is a barrier among immigrants who are undocumented [31]. In some US states and Canadian provinces, youth are required to obtain the consent of their parents or legal guardians and need to be a certain age in order to apply for identification. For instance, in Ontario, youth have to be at least thirteen years of age to apply for many forms of PID on their own behalf, and for youth who are minors and estranged from parents or guardians, age-related restrictions present significant barriers [25,32] and potential danger for those individuals trying to avoid foster care or the return to a less than safe environment. Young women who are homeless reported facing judgement and censure from health care providers [28]. One study also reported stigmatizing attitudes towards people who were homeless in general [18]. For female sex workers in Miami, a lack of space for the storage of identification posed a problem; without storage space for possessions, “women are often assaulted or otherwise robbed of the few goods they own,” including their IDs [33] (p. 353). In some Canadian provinces (e.g., Ontario, British Colombia, New Brunswick), there is a three-month waiting period for a provincial health card for newcomers [34], leaving people in a vulnerable position should they require emergency services during the window of no coverage.

A few studies (10%, *n* = 3) also reported barriers in the accurate and complete reporting of personal information, like date of birth and the incorrect recording of names and place of birth [35,36,37]. A study by Melnik and colleagues explored the accuracy of birth data collected in New York State facilities, and found barriers including incomplete information provided by medical staff, birth data located in multiple systems, conflicting birth data from different sources, and inadequate staff resources [36]. In California, Smith and colleagues found the misclassification of ethnicity and race in administrative records in 23.1% and 33.6% of children, respectively [37]. The authors reported two major causes of this misclassification, including missing information in administrative records and the classification of children of multiple races based on information from only one parent.

While many studies (55%, *n* = 17) included the socio-demographic characteristics of participants, such as age, gender, and ethnicity [18,38,39], few attempted to differentiate people’s experiences and perspectives that result from these characteristics. For example, 385 adults in Toronto who were homeless that participated in a survey included, but were not limited to, 63% white, 12% black, and 9% Indigenous [38]. However, while the study found that 34% of participants had a health card, it did not indicate whether this outcome corresponded with a particular racial identity. Information on which ethnic groups possessed a health card would help inform more nuanced efforts to increase access to identification and health care more generally. A notable exception where this information was included is a qualitative study in Edmonton, where 40% of interviewees (*n* = 41) were estimated to be Indigenous, with the majority being men [27]. The study found that Indigenous men and women experienced more barriers to identification on average compared to non-Indigenous men and women. In a different study from California, Smith and colleagues found that children of minority groups are more likely than non-minority groups to experience the misclassification of ethnicity and race in administrative records, presenting possible consequences for data misinterpretation and over/underestimated health disparities, as well as presenting further difficulties later in life if and when people have to replace their PID [37].

The challenge posed by PID was further exacerbated for sexual minorities, particularly transgender individuals [25,39]. In a study exploring the lived experiences of 27 transgender youth that were homeless in New York City, many either did not have identification or had identification documents that did not match their self-designated gender and presentation, resulting in “transgender and gender expansive young people facing harassment and discrimination when applying for jobs” [39] (p. 16).

### 3.2. Access to Health and Social Services

Following from the inability to acquire or maintain PID are the social and health consequences that directly result. The lack of identification was reported by many studies (42%, *n* = 13) as a factor impacting the ability of individuals to access health services (e.g., [17,40,41]). For example, one provider in Calgary, Canada reported:

*Identification is something that you often need when you go to clinics and a lot of our [clients] do not have ID—whether or not they even have Alberta Health Care cards with them or have even applied for their Alberta Health Care cards. We have a lot of out-of-province clients that come through, a lot of immigrants that come through so then that whole issue is do they even get access to certain types of care just due to not having the proper documents [17] (p. 7).*

A lack of PID becomes both a direct and indirect barrier to accessing services. In Ontario, for example, residents must present an Ontario health card in order to receive benefits through provincially funded health coverage [24]. To receive a health card, however, an individual must provide three key documents (proof of citizenship, proof of Ontario residency, and some form of personal identification from a specified list), which poses significant difficulties for people with precarious housing. Bureaucratic structures with onerous requirements for applying for PID can further complicate matters for many people. In a qualitative study involving 54 youth in Los Angeles who were homeless and drug-dependent, the authors reported:

*Perhaps surprisingly, structural barriers cited by the youth stemmed not from a paucity of agencies or resources but conversely from the presence of too many agencies with endless bureaucratic requirements involving interagency referrals, the need for identification cards, time-consuming paperwork, and lack of continuity of care [42] (p. 158).*

This was also echoed in a qualitative study involving 20 young women in Seattle experiencing homelessness:

*So you have to go to a regular clinic and they take forever to register you and they want to know why you don’t have insurance and then they make you sit there another 45 minutes until they call someone to figure out what it is. I’ve had so many bills from places like that so many notices. I always told them from the beginning, ‘I’m homeless. I don’t have an ID. You can’t call my parents; they will not say they’re my guardians. They will not take responsibility for me. I don’t have insurance.’ You know—it’s like, ‘Can you please? I’m bleeding here - can you help me’? [28] (p. 169).*

According to some studies (26%, *n* = 8), government-issued identification is also required to access food banks (e.g., [19,20,26]). A survey of service providers across 16 US states found that when individuals who were homeless could not provide photo identification, 53% were denied food stamps [29]. Another survey of homeless adults in downtown Toronto reported that 15% of adults that were homeless were unable to access the food bank due to a lack of identification [43]. In New York City, 29 out of the 47 (62%) food pantries surveyed had an identification requirement [44].

In an unnamed city in the US, individuals living with mental health disabilities and facing homeless were found to face further challenges to accessing services as a result of the lack of PID:

*Returning offenders who have mental illness are often eligible for several public assistance programs, including General Assistance, food stamps, and Medicaid. In the state where the study site is located, all such programs are administered by the state’s public assistance department, which also oversees the application process and thereby controls access to services. Identification requirements are a central feature of the application process, and these requirements emerged early in the study as a source of problems for clients” [19] (p. 117).*

Indeed, a lack of PID was identified by several studies (13%, *n* = 4) as a serious barrier to accessing social housing and income support (e.g., [43,45,46]). For example, the lack of personal identification was reported as a barrier of many people who were either homeless or precariously housed that were applying to the Ontario Disability Support Program [46]. In a survey of 368 adults in Toronto that were homeless, 22 (6%) reported that the lack of PID was the main reason for remaining homeless [43].

Suggestions for reducing barriers to accessing health and social services include: welcoming other forms of identification (e.g., non-government issued identification) [20], providing alternative verification processes for proof of identity or residence (e.g., allowing people who were homeless to use the address of a shelter as their mailing address) [25,32], building mechanisms to improve access to services that do not require individuals to present identification (e.g., databases that transfer medical data between sites) [38,42], building the cultural competencies of health care providers [18,28,33,47], and improving the access and availability of information on how to obtain identification and reducing or eliminating fees [31,32].

### 3.3. Facilitating Identification Programs and Services

Finally, a number of studies exploring PID facilitators (13%, *n* = 4) recommended funding programs at social service agencies to support the replacement and storage of identification [25,38,48,49]. Kopec and Cowper-Smith described four organizations in Canada that provide a space to store identifications (sometimes referred to as “ID banks”) [50]. Most of these organizations also help clients apply for their identification and cover the associated fees. Similarly, Goldblatt and colleagues described two identification programs that provide a mix of support services at no fee [45]. In one case, the Regional Municipality of York Region in Ontario provides a mailing address for clients when necessary, delivers identification to clients, and connects individuals with other services such as housing, food resources, and financial support. In the second case, the city of Toronto provided funding to support the ID Bank located at Street Health. Other studies argued that ID fees for people who were homeless should be waived [29,30,31,32]. In one state, South Carolina, people that were homeless were not required to pay fees associated with PID:

*In order to get a fee waiver, a homeless person provides a letter from a shelter employee or other service provider indicating that he is homeless and requesting a fee waiver [29] (p. 21).*

## 4. Discussion

Within the modern bureaucratic state, personal identification serves many, often contradictory, purposes. On the one hand, establishing identity can connect individuals to vital health and social services, while, on the other hand, the theft and misuse of identity can have devastating consequences, ranging from breaches in personal privacy and financial fraud to the loss of democratic freedoms when governments use personal data to surveil individuals and populations. Recent conversations about PID have tended to focus on the latter issues, precipitated mainly by the events of 9/11 and recent high-profile cases of cybertheft and the sale of personal data by major corporations. While it is important to acknowledge the validity of these concerns, this scoping review focuses on the former issue by drawing attention to the central role played by PID in accessing essential state services, particularly among the most socially and economically marginalized people and groups in society.

We started this research prior to the outbreak of, and public health response to, the COVID-19 global pandemic, an event that makes it more apparent than ever how a lack of PID impacts access to the social determinants of health for the most marginalized people in society. At the time writing, local emergency food banks require valid identification, not only for the individual directly receiving the food, but for everyone living in the household [51,52,53]. Many people simply do not have access to PID documents and information at this tumultuous time, let alone are they able to afford the cost of a PID application at the moment. Marginalized people without PID are unable to travel home by air or bus, nor can they access many emergency housing supports, as these options all require PID, leaving some with no alternative but to live on the streets where physical distancing and other protective measures, like hand-washing, cannot be practiced [54]. Government service centers that normally process PID applications have limited both their business hours and their provision of services [55], and while these measures are important in helping to flatten the curve of COVID-19, they also further marginalize people in need of emergency services by making it exceedingly difficult to obtain PID at a time when it is needed most.

The results of this scoping review illustrate the paucity of research on what may be termed the “problem of personal identification,” especially in regards to the barriers and facilitators faced by groups that are particularly marginalized in the acquisition and retention of PID. Our review also finds that the existing research, while limited, focuses primarily on people who are either homeless or precariously housed; to a lesser extent, the review also finds that sex workers and select sexual minorities face significant PID challenges, namely transgender people. It is also worth pointing out that almost one-third of our results come from the gray literature, in the form of reports and policy briefs produced by nonprofit organizations, like Street Health in Toronto, Canada. This suggests that a significant portion of the work on PID is being conducted by frontline organizations and that more academic involvement could support these organizations to study the issue more comprehensively.

Among the most common barriers to PID, the scoping review finds that homelessness creates obstacles to the acquisition of PID, as often an address is required to apply for PID, as well as to maintaining the possession of PID, as theft of and damage to personal belongings is an ever-present problem. Another key barrier associated with a lack of PID is an inability to access social and health services, which, in turn, makes people who are marginalized further vulnerable by limited access to the social determinants of health; this problem is particularly marked among women and youth. Finally, regarding facilitators, the review finds that identification programs, such as “ID banks,” are positively associated with people’s ability to acquire and maintain PID. These findings highlight important sociological interactions, ranging from economic deprivation and homelessness to gender and sexual identity, that contribute to people’s ability to acquire or maintain key forms of PID that are the gateway to accessing vital services.

Another notable finding of the scoping review was a pointed statement shared by several interviewees of one study: “You need ID to get ID…you can’t do anything without ID” [27] (p. 256). This reality speaks to the importance of birth registration and maintaining the possession of a birth certificate. In Canada, for instance, a birth certificate is required to acquire most forms of identification, such as a Social Insurance Number (SIN) or an Indian Status Card, which is required under the Indian Act to confirm the Indian status of Indigenous people. Even for forms of PID that do not directly require a birth certificate, such as an Ontario health card or driver license, a birth certificate is necessary to get the prerequisite identification needed to apply for a health card or driver license. Thus, in Canada, as in many other nations, the birth certificate becomes the foundational piece of PID that enables access to all other identification documents. That many PID applications require a permanent residence in order to be issued becomes a “catch 22” situation of sorts, wherein people who are precariously housed require a home in order to obtain PID that will enable them to access housing or health and social services.

Conspicuously absent from the existing literature was research that focused on northern and rural populations, Indigenous people, and the relationship between the two. In Canada, for instance, Indigenous people make up a significant proportion of the population in the rural and provincial north, and further clarity is needed on the unique PID problems facing this population, such as birth registration and the acquisition of birth certificates, as well as the difficulties of obtaining PID in areas with extremely limited access to state social and health services [6]. Our preliminary work, for example, has shown that 73% of the clients seeking birth certificates and other forms of PID in Thunder Bay and the surrounding district identify as Indigenous [56], indicating that this is an important area of further study. Likewise, the structural barriers that exist in fly-in and road access First Nations have not been addressed, nor is there any sustained analysis of the historical and ongoing impacts of settler colonialism on access to and the meaning of PID.

Although a few studies identified the reporting of inaccurate birth information by medical staff or other administrative personnel as a barrier to acquiring PID, the particular experiences of Indigenous peoples in the north and the implications have not yet been fully fleshed out. For instance, Indigenous children forced to attended residential schools frequently had their names changed, misspelt, or dates of birth recorded incorrectly [57]. Records of these activities, which would help substantiate claims of identity, have often been lost to fires and flooding that frequently occur in rural settings. That this is a historical problem, dating back to the 1800s, means that elders from rural areas are even less likely to have access to original documentation required to acquire PID. Furthermore, these problems have persisted for Indigenous children, who continue to be removed from their families and communities at alarming rates by child welfare agencies. In Canada, Indigenous children account for 48% of the children in foster care, while constituting only 4.3% of the Canadian population [58]. The fear of possible child apprehension may also pose a further barrier to birth registration for Indigenous peoples, if parents are afraid to report new births [59,60]. Long histories of settler violence enacted through systems of education, health care, policing, and child welfare have ensured that Indigenous people and communities have been over-policed and under-serviced by the state. As a result, mechanisms, like birth certificates and other forms of PID, which make citizens visible to state structures and services, can often be problematic and fraught with anxiety and distrust for Indigenous people.

More research is also needed on the implementation and use of “ID banks” as a facilitator for acquiring and maintaining PID. Storage programs are particularly promising for people who are homeless, especially as the conditions of living unhoused frequently leads to the damage and permanent loss of PID [61,62]. Such programs exist in different forms in urban areas, offering a variety of storage options for PID. Options include the storage of original copies of PID, official duplicates, and unofficial photocopies, as well as the storage of digital copies on secure servers. In some instances, an unofficial photocopy of PID may be adequate to prove personal identity or, at least, to begin the process of applying for certain services contingent upon the client returning with the original identification document to complete the process. In other cases, agencies that host ID banks can also be contacted to vouch that the photocopy is accurate and on file; this model can be particularly effective among partnering agencies or those with a memorandum of understanding (MOU) for specific issues.

Importantly, there are examples of agencies that work with people who are homeless to create their own form of “agency ID card” for clients, which is recognized by local law enforcement due to the agency’s reputation (e.g., Street Health in Toronto, ON, Canada). Furthermore, some agencies with ID banks have staff with registered notary status, enabling them to make notarized copies of PID on site. A staff member who can serve as a notary alleviates one more complicated, if not costly, step, as notary services can be prohibitive for people who are economically disadvantaged. Using an ID bank service means that clients know their PID is safely stored and can be accessed during agency business hours (or whatever access schedule is in place). Some agencies also serve as a mailing address where clients may have identification documents sent for official receipt and safe storage. ID banks may be one way that frontline service agencies with extremely limited resources can begin to address the PID problem among their clientele. Research on this topic should focus on the structure and design of ID banks, common/best practices, who uses them and why, which agencies have established them and to what effect, and barriers to implementation. It is also important to further explore the ways in which different national and provincial/state jurisdictions and policies affect the implementation and design of ID banks. If the process of instituting an ID bank is too costly or bureaucratically onerous, many community agencies with limited resources will be deterred from attempting to provide this important service. Finally, it is important to better understand the implementation and use of ID banks in rural areas, as the current literature deals exclusively with urban settings.

### Study Limitations and Future Directions

It is important to consider the potential risk of bias within this review. First, this scoping review was limited to English-language articles, which most obviously biases findings toward higher income Western nations but also, in the case of Canada, excluded francophone areas like Quebec. While many of the themes identified in the literature are likely national and therefore also exist in Quebec, PID barriers and facilitators that are particular to that province require further investigation prior to the development and implementation of federal policy. Second, in general scoping reviews, including this one, do not evaluate the methodological quality of the studies nor the quality of the evidence, but rather focus more broadly on the outcomes presented by the studies [63]. Third, a further limitation of this study was the decision to limit the scope of analysis to PID in North America. This decision was anchored in our particular research project that examines the PID experiences of Indigenous people in Canada and the US—nations that have similar policies and practices. Undoubtedly, expanding the scope of the analysis to include places like Europe and Australia, for example, would shed additional valuable light on the experiences of other marginalized groups, including ethnic minorities and refugee and migrant communities, as well as the bureaucratic practices of other nations with respect to PID. Finally, as with any scoping review, some literature may have been missed as a result of the keyword search strategy and the limitations of the selected databases, which may, for instance, limit the ability to locate key gray literature. The Google search alone, for example, might not capture all of the relevant gray literature [15]. For a more comprehensive analysis, future analyses might look at websites of key organizations or contact organizations to inquire if they have unpublished sources available. Nevertheless, this scoping review is rigorous and provides insights into some of the PID key barriers and important facilitators in North America.

## 5. Conclusions

This scoping review is the first step toward investigating the problem of PID through an intersectional lens. Our findings indicate that PID is an important influence on the ability of people who are marginalized to acquire and maintain PID that, among other things, enables access to the social determinants of health. It is our position that a more complete understanding of the barriers and facilitators to PID is imperative, particularly in different local, regional, and national contexts, as well across a diverse range of social identities. Such research will benefit multiple disciplines in the social and health sciences and nursing, as well as policy-oriented fields. Interweaving this understanding with a more sophisticated understanding of the social determinants of health would further highlight ways that poverty and social factors, like racism and colonialism, help reproduce one another. This would not only provide a more nuanced understanding of the problem of PID, but contribute to evidence-informed policy aimed at ameliorating the problem and improving health outcomes among people that are the most underserved and marginalized in society.

## Figures and Tables

**Table 1:** Search strategy to identify peer-reviewed articles on barriers and facilitators to obtaining identification.

Main Terms	Expanded Terms
Identification	(“Photo identification” OR “personal identification” OR “government-issued identification” OR “civil identification” OR “birth identification” OR “birth certificat *” OR “birth registration” OR “photo ID”) AND
Barriers and facilitators	((barrier * OR challenge *) OR (facilitator * OR opportunit *))

* Boolean operator symbol for truncation used to broaden search by capturing all variations of words.

**Table 2:** Summary of the descriptive characteristics of 31 studies on the barriers to and facilitators of obtaining identification in North America.

Characteristics	No.	%
Document type:		
Peer-reviewed journal article	22	71.0
Research report	9	29.0
Study location:		
Canada	16	51.6
USA	15	48.4
Study scale:		
Local	25	80.6
State/provincial wide	4	12.9
Not specified	2	6.5
Approach:		
Qualitative	14	45.2
Quantitative	9	29.0
Other (not specified, theoretical)	6	19.4
Mixed qualitative quantitative	2	6.4
Target population ^a^:		
Homeless people in general	11	12.9
Homeless youth specifically	4	9.7
Homeless adults specifically	3	9.7
General population	3	6.5
Sexual minorities	2	6.5
Sex workers	2	6.5
Injection drug users	2	6.5
Immigrants	2	6.5
Refugees	1	3.2
Previous offenders	1	3.2
Children	1	3.2
Cancer patients	1	3.2

^a^ Numbers do not add up because target population could have multiple characteristics.

## Appendix A. Details for Relevant Findings in Reviewed Literature

**Academic Literature on PID**					
**No.**	**Author (Year)**	**Country**	**Approach/Method**	**Objective**	**Relevant Findings**
1	Appel et al. (2004) [40]	USA	Qual/interviews with outreach staff (*n* = 55) and intravenous drug user (IDU) clients (*n* = 144)	Obstacles to AOD (alcohol & other drug) enrollment inherent in policies, practices, and attitudes of institutions	Lack of ID among top five barriers to enrollment and completion of drug abuse treatment program; client suggestions for program facilitators include “admit people who have no PID or documents”
2	Asanin et al. (2008) [34]	Canada	Qual/focus groups (*n* = 8) with 58 immigrants from diverse race/ethnic backgrounds	Explore immigrants’ perceptions of barriers to access to healthcare	Delay in receiving provincial health card results in either lack of healthcare access for several months or in paying for private health insurance, which is costly for many recent immigrants
3	Butters et al. (2003) [24]	Canada	Qual/interviews with female street-involved crack users (*n* = 30)	Examine healthcare needs and experiences women who were involved in the street life of crack and prostitution	ID a key factor affecting access to health care; to get a health card you need ID and an address (which homeless people do not have); 43% did not have a health card for reasons including never had one, lost, stolen, or destroyed
4	Campbell et al. (2015) [17]	Canada	Qual/interviews and focus groups with homeless individuals (*n* = 11) and healthcare providers (*n* = 11)	Explore perceived healthcare needs and barriers among individuals experiencing homelessness	Many providers and clients described that an important barrier to accessing healthcare services is lack of government-issued ID, including provincial healthcare card; missing/stolen ID cited as common problem
5	Cheng et al. (2016) [35]	USA	Quant/analysis of electronic database (*n* = 11,989)	Evaluate associations of missing paternal demographic information on birth certificates with perinatal risk factors for childhood obesity	Six percent were missing paternal data; missing paternal data on the birth certificate is associated with perinatal risk factors for childhood obesity
6	Christiani et al. (2008) [42]	USA	Qual/focus groups with homeless drug-using youth (*n* = 54)	Investigate barriers and facilitators in delivery of healthcare services from the perspectives of homeless youth	Structural barriers include agencies with multiple bureaucratic requirements involving interagency referrals, need for ID cards, onerous paperwork, and lack of continuity of care
7	Daiski (2005) [41]	Canadian	Qual/interviews and focus groups with client volunteers (*n* = 58)	Evaluate effectiveness of the Health Bus, a mobile healthcare unit	At one location, only half of the participants possessed a health card. Health cards and other ID often stolen or lost; the Health Bus was the only healthcare people without a health card accessed; another barrier is attitude of health care workers
8	Ensign et al. (2002) [28]	USA	Qual/interviews with homeless young women (*n* = 20)	Explore common sources of advice, health-seeking behaviors, and access to care issues of homeless adolescent women	Participants said that the biggest structural barriers to care at many hospitals or clinics not designed for homeless youth were questions over consent for care, being asked to provide addresses and an ID card, and source of insurance or payment
9	Gany et al. (2013) [44]	USA	Quant/assessment survey administered in-person to randomly selected food pantries (*n* = 47)	Analyze how well food pantries accommodate the service needs of medically ill clients	Twenty-nine food pantries (62%) had an identification requirement, including six (or 21%) requiring a government-issued photo ID; requirements for government-issued ID likely to discourage undocumented immigrants
10	Gordon (2012) [27]	Canada	Qual/interviews with 102 people visiting ID clinic established to provide basic ID card provided by municipality free of charge	Explore reasons that people lack ID, impact of no ID, and significance that new ID card has for them	Main reasons for ID loss: theft, carelessness, police interaction, drug/alcohol event; main impact of no ID: banking, healthcare, housing, alcohol/cigarette purchase, police checks, treaty benefits, driving, money mart usury; significance of new ID: member of community, access to services
11	Heyman (2009) [31]	USA	Mixed/survey of uninsured immigrants (*n* = 84); interviews with same (*n* = 52)	Determine healthcare-seeking paths by unauthorized migrants, barriers encountered, and resilience factor of learning and gaining confidence about available services	Participants reported increased demands for PID in order to get healthcare (e.g., employment history, social security number, and citizenship or legal residence ID. “Ernesto has never had these documents…but this documentation requirement is now a basis for denial of services that he previously received.” He remarked, “They [the hospital] are asking for things I cannot produce” (p. 11)
12	Hwang et al. (2000) [48]	Canada	Quant/document review of patient encounter and billing records (*n* = 45)	Document how frequent physicians are not paid for the care they provide to homeless people in outreach settings	Study finds high proportion of homeless people without valid health insurance cards; 46% of encounters with patients without a health card at all, though reasons not specified
13	Hwang et al. (2010) [18]	Canada	Quant/survey of homeless individuals (*n* = 1169)	Examine extent of unmet needs and barriers to accessing healthcare among homeless people within a universal health insurance system	Three hundred and fifteen participants were not in possession of their health insurance card; often it was lost or stolen
14	Khandor et al. (2011) [38]	Canada	Quant/survey of homeless adults (*n* = 385)	Examine active healthcare-seeking paths of unauthorized migrants, range of barriers that they encounter, and resilience factor of learning and gaining confidence about available services	One hundred and twenty-four (34%) participants were not in possession of a health card; most of the people without a health card reported that their card had been lost (48%) or stolen (18%)
15	Kurtz et al. (2005) [33]	USA	Mixed/surveys of street-based female sex workers (*n* = 586); focus groups with same (*n* = 25)	Assess health and social service needs and barriers to access among street-based women sex workers	Lack of legal ID, address, and/or citizenship status causes women to be ineligible for most employment. Lack of space for ID storage is also a problem, as sex workers are often robbed
16	LeBrón et al. (2018) [20]	USA	Theoretical/not stated	Describe three ways government-issued ID requirements have restricted access to health resources	An estimated 11% of voting-age US citizens lack ID, with certain communities additionally marginalized due to lacking a permanent address (e.g., people who are homeless or experienced catastrophic events like fire, domestic violence, environmental disaster)
17	McKeary et al. (2010) [47]	Canada	Qual/interviews with healthcare providers (*n* = 14)	Explore the systemic barriers to healthcare access experienced by refugee populations	Lack of insurance coverage also created a barrier to care (6.3%) six months after arrival, but this disappeared with access to a provincial health card granted after residency waiting period. Additional ID barriers include burdensome paperwork, unclear eligibility rules, poverty, isolation, lack of access to transportation, language, and illiteracy
18	Melnik et al. (2015) [36]	USA	Quant/survey of birth registrars (*n* = 127)	Better understand the current birth registration practices and barriers in accurate and complete reporting	Incomplete information provided by medical staff, birth data are located in multiple systems, conflicting birth data information from different sources, staff resources are inadequate, need for improvement of hospital electronic data systems
19	Pauly (2008) [64]	Canada	Theoretical/not stated	Examine harm reduction values and conceptions of justice as framework for addressing inequities in health and healthcare for street-involved drug users	Lack of ID is barrier for accessing health care services or gaining prescription coverage
20	Shelton (2015) [39]	USA	Qual/interviews with young people (*n* = 27)	Investigate the lived experiences of transgender and gender expansive young people with histories of homelessness	Some participants arrived at shelters lacking ID; without ID documents that match their self-designated gender and presentation, transgender and gender expansive young people face harassment and discrimination when applying for jobs. Lack of accurate ID increases likelihood they will experience misgendering during hiring process and while on the job, if hired. Frequent experiences of cisgenderism lead some to not seek employment
21	Smith et al. (2010) [37]	USA	Quant/comparison of health plan records with hospital birth certificate records of 325,810 children born between 1998–2008	Investigate validity of race and ethnic information in health plan administrative records and if this affects understanding of race/ethnic disparities in healthcare utilization	Birth certificates have more accurate data on parental status at time of birth and race/ethnicity; easier access to birth certificates for birth information will improve health access and health outcomes; inconsistent PID information presents difficulties later in life if people need to replace PID
22	Wilson (2009) [19]	USA	Mixed/interviews with staff (*n* = 10) and clients (*n* = 14) of prisoner re-entry program; document review; ethnographic observation	Examine range of help-seeking activities; identify dynamics that facilitate access to public services for recently incarcerated people re-entering society	Lack of ID is key barrier for returning offenders; ID often lost during arrest and incarceration or due to mental health challenges. Without ID it is difficult for offenders to apply for ID, a cycle that leads to delays in access to health and social services for people trying to re-enter society and get jobs
**Gray Literature on PID**					
**No.**	**Author (Year)**	**Country**	**Approach/Methods**	**Objective**	**Relevant Findings**
23	Goldblatt et al. (2011) [45]	Canada	Qual/interviews with staff from social housing support organizations (*n* = 48)	Identify systematic barriers to housing experienced by service providers	Lack of ID is key barrier to accessing social housing and income support
24	Hussey (2015) [25]	USA	Not stated	Identify structural barriers that LGBT youth face in attaining ID and identify policy recommendations for overcoming barriers	Barriers include age restrictions, fees, lack of address, as well as unique barriers for certain communities (e.g., transgender youth, undocumented youth, system involved youth). Recommendations include: establish municipal ID programs that can be tailored to community needs; reduce or waive fees for ID; easing parental approval for ID for a minor, as many LGBTQ youths experience homelessness due to family rejection
25	Kopec et al. (2016) [50]	Canada	Qual/interviews with staff at four ID banks	Explore avenues available for creating an ID bank for homeless and vulnerable clients	Main barriers are funding and staffing, though once established, ID storage can be inexpensive
26	NLCHP (2004) [29]	USA	Quant/survey of service providers (*n* = 56) in 16 states that serve total of 25,647 clients per year	Assess nature and extent of the problems faced by homeless persons without photo ID	Around one-tenth (10.7%) of clients lacked photo ID; barriers include lack of address and high cost/fees; implications of not having ID include: denied access to benefits and services and problems with law enforcement
27	NNY (2016) [32]	USA	Not stated	Provide overview of issues related to minors obtaining an ID and related recommendations for policy makers	Four main barriers to obtaining ID: needing/obtaining birth certificate, age limits and parental consent, fees, and proof of residency
28	Shepherd et al. (2003) [49]	Canada	Not stated	Provide updated information on state of poverty, housing, and homelessness in Toronto	Homeless people at greater risk of having ID, such as their health card, stolen or lost; yet they are most vulnerable and likely to require healthcare
29	Shartal et al. (2004) [46]	Canada	Qual/interviews with homeless people with disability (*n* = 85)	Describe experiences of homeless people with disabilities unable to access Ontario Disability Support Program (ODSP)	Homeless people often do not possess ID and financial documents required to apply for the program; thus, ID is structural barrier for most marginalized people
30	The Street Health Report (2007) [43]	Canada	Quant/survey of homeless adults (*n* = 368)	To fill a gap in current knowledge about the health status of homeless people in Toronto	Six percent of respondents said lack of ID is reason for homelessness; ID lost or taken away by authorities; importance of ID for accessing social services
31	United Way (2013) [26]	Canada	Not stated	Not stated	Barriers include cost and need of address to get ID

## References

[1] Harper J. (2006). Identity Crisis: How Identification is Overused and Misunderstood.

[2] Bennett C.J., Lyon D. (2008). Playing the Identity Card: Surveillance, Security and Identification in Global Perspective.

[3] Rygiel K., Rygiel K., Hunt K. (2008). Protecting and Proving Identity: The Biopolitics of Waging War through Citizenship in the Post-9/11 era. (En) Gendering the War on Terror: War Stories and Camouflaged Politics.

[4] Taylor J.A., Lips M., Organ J. (2008). Identification practices in government: Citizen surveillance and the quest for public service improvement. Identity Inf. Soc..

[5] Pais M.S. (2002). Editorial: Birth Registration: Right from the Start. Innocenti Dig..

[6] Sanders C., Burnett K. (2019). A case study in personal identification and social determinants of health: Unregistered births among Indigenous people in northern Ontario. Int. J. Environ. Res. Public Health.

[7] Lorde A. (2007). Sister Outsider: Essays and Speeches.

[8] Kulig J.C., Williams A.M. (2012). Health in Rural Canada.

[9] Adelson N. (2005). The embodiment of inequity: Health disparities in Aboriginal Canada. Can. J. Public Health.

[10] Gracey M., King M. (2009). Indigenous health part 1: Determinants and disease patterns. Lancet.

[11] Parkins J.R., Reed M.G. (2013). Social Transformations in Rural Canada: Community, Cultures, and Collective Action.

[12] Arksey H., O’Malley L. (2005). Scoping studies: Towards a methodological framework. Int. J. Soc. Res. Methodol..

[13] Colquhoun H.L., Levac D., O’Brien K.K., Straus S., Tricco A.C., Perrier L., Kastner M., Moher D. (2014). Scoping reviews: Time for clarity in definition, methods, and reporting. J. Clin. Epidemiol..

[14] Tricco A.C., Lillie E., Zarin W., O’Brein K.K., Colquhoun H., Levac D., Moher D., Peters M.D.J., Horsley T., Weeks L. (2018). PRISMA extension for scoping reviews (PRISMA-ScR): Checklist and explanation. Ann. Intern. Med..

[15] Godin K., Stapleton J., Kirkpatrick S.I., Hanning R.M., Leatherdale S.T. (2015). Applying systematic review search methods to the grey literature: A case study examining guidelines for school-based breakfast programs in Canada. Syst. Rev..

[16] Braun V., Clarke V., Hayfield N., Terry G., Liamputtong P. (2018). Thematic analysis. Handbook of Research Methods in Health Social Sciences.

[17] Campbell D.J.T., O’Neill B.G., Gibson K., Thurston W.E. (2015). Primary healthcare needs and barriers to care among Calgary’s homeless populations. BMC Fam. Pract..

[18] Hwang S.W., Ueng J.J.M., Chiu S., Kiss A., Tolomiczenko G., Cowan L., Levinson W., Redelmeier D.A. (2010). Universal health insurance and health care access for homeless persons. Am. J. Public Health.

[19] Wilson A.B. (2009). It takes ID to get ID: The new identity politics in services. Soc. Serv. Rev..

[20] LeBrón A.M.W., Lopez W.D., Cowan K., Novak N.L., Temrowski O., Ibarra-Frayre M., Delva J. (2018). Restrictive ID policies: Implications for health equity. J. Immigr. Minor. Health.

[21] Evans R.D., Forsyth C.J. (2004). Risk Factors, Endurance of victimization, and survival strategies: The impact of the structural location of men and women on their experiences within homeless milieus. Sociol. Spectr..

[22] Daiski I. (2007). Perspectives of homeless people on their health and health needs priorities. J. Adv. Nurs..

[23] Novac S., Hermer J., Paradis E., Kellen A., Hulchanski J.D., Campsie P., Chau S., Hwang S., Paradis E. (2009). More Sinned Against than Sinning? Homeless People as Victims of Crime and Harassment. Finding Home: Policy Options for Addressing Homelessness in Canada (E-Book).

[24] Butters J., Erickson P.G. (2003). Meeting the health care needs of female crack users: A Canadian example. Women Health.

[25] Hussey H. (2016). Expanding ID Card Access for LGBT Homeless Youth.

[26] United Way (2013). Policy Brief on Government Identification.

[27] Gordon R. (2012). Community, use it or lose it?. Anthropologica.

[28] Ensign J., Panke A. (2002). Barriers and bridges to care: Voices of homeless female adolescent youth in Seattle, Washington, USA. J. Adv. Nurs..

[29] National Law Center on Homelessness & Poverty (2004). Photo Identification Barriers Faced by Homeless Persons: The Impact of September 11.

[30] The United Nations Children’s Fund (UNICEF) (2005). The ‘Rights’ Start to Life: A Statistical Analysis of Birth Registration.

[31] Heyman J.M.C., Núñez G.G., Talavera V. (2009). Healthcare access and barriers for unauthorized immigrants in El Paso County, Texas. Fam. Community Health.

[32] National Network for Youth (2016). A State-By-State Guide to Obtaining ID Cards.

[33] Kurtz S.P., Surratt H.L., Kiley M.C., Inciardi J.A. (2005). Barriers to health and social services for street-based sex workers. J. Health Care Poor Underserved.

[34] Asanin J., Wilson K. (2008). ‘I spent nine years looking for a doctor’: Exploring access to health care among immigrants in Mississauga, Ontario, Canada. Soc. Sci. Med..

[35] Cheng E.R., Hawkins S.S., Rifas-Shiman S.L., Gillman M.W., Taveras E.M. (2016). Association of missing paternal demographics on infant birth certificates with perinatal risk factors for childhood obesity. BMC Public Health.

[36] Melnik T.A., Guldal C.G., Schoen L.D., Alicandro J., Henfield P. (2015). Barriers in accurate and complete birth registration in New York state. Matern. Child Health J..

[37] Smith N., Iyer R.L., Langer-Gould A., Getahun D.T., Strickland D., Jacobsen S.J., Chen W., Koebnick C. (2010). Health plan administrative records versus birth certificate records: Quality of race and ethnicity information in children. BMC Health Serv. Res..

[38] Khandor E., Mason K., Chambers C., Rossiter K., Cowan L., Hwang S.W. (2011). Access to primary health care among homeless adults in Toronto, Canada: Results from the street health survey. Open Med..

[39] Shelton J. (2015). Transgender youth homelessness: Understanding programmatic barriers through the lens of cisgenderism. Child Youth Serv. Rev..

[40] Appel P.W., Ellison A.A., Jansky H.K., Oldak R. (2004). Barriers to enrollment in drug abuse treatment and suggestions for reducing them: Opinions of drug injecting street outreach clients and other system stakeholders. Am. J. Drug Alcohol. Abuse.

[41] Daiski I. (2005). The health bus: Healthcare for marginalized populations. Policy Polit. Nurs. Pract..

[42] Christiani A., Hudson A.L., Nyamathi A., Mutere M., Sweat J. (2008). Attitudes of homeless and drug-using youth regarding barriers and facilitators in delivery of quality and culturally sensitive health care. J. Child Adolesc. Psychiatr Nurs..

[43] Hulchanski J.D., Campsie P., Chau S., Hwang S., Paradis E., The Street Health Report (2007). The Street Health Report. The Health of Toronto’s Homeless Population. Finding Home: Policy Options for Addressing Homelessness in Canada (E-Book).

[44] Gany F., Bari S., Crist M., Moran A., Rastogi N., Leng J. (2013). Food insecurity: Limitations of emergency food resources for our patients. J. Urban Health.

[45] Goldblatt A., Felix R., Chotai V., Fleger M. (2011). Final Report: Systemic Barriers to Housing Initiative.

[46] Shartal S., Cowan L., Khandor E., German B. (2004). Failing the Homeless: Barriers in the Ontario Disability Support Program for Homeless People with Disabilities.

[47] McKeary M., Newbold B. (2010). Barriers to care: The challenges for Canadian refugees and their health care providers. J. Refug. Stud..

[48] Hwang S.W., Windrim P.M., Svoboda T.J., Sullivan W.F. (2000). Physician payment for the care of homeless people. CMAJ.

[49] Shepherd S., Low H., Meisner A. (2003). The 2003 Toronto Report Card on Housing and Homelessness.

[50] Kopec A., Cowper-Smith Y. (2016). Guelph-Wellington Taskforce for Poverty Elimination: Avenues for Creating an ID Bank.

[51] Lazaruk S. (2020). Greater Vancouver food-bank users will soon need to prove low-income status. Vancouver Sun.

[52] Linton B. (2020). Food bank deals with location change. The Chronicle Journal.

[53] Pickrell A. (2020). Sudbury food bank updates guidelines for new users. CTV News.

[54] Hamilton W. (2020). Can’t go home: No ID strands Indigenous man on Vancouver’s Downtown Eastside. CBC.

[55] Silcoff S., Cardoso T. (2020). Ottawa shuts Service Canada centres after employees refuse to work. The Globe and Mail.

[56] Burnett K., Sanders C., Skinner K. The Challenges of Accessing Personal Identification in Northwestern Ontario. Proceedings of the Keynote Address presented to Kinna-aweya Legal Clinic Annual General Meeting.

[57] National Centre for Truth and Reconciliation (NCTR) (2015). National Centre for Truth and Reconciliation.

[58] Statistics Canada (2013). Aboriginal Peoples in Canada: First Nations People, Métis and Inuit, Part 3 Living arrangements of Aboriginal Children. National Household Survey, 2011.

[59] Canadian Broadcasting Corporation (CBC) Province Reports 1st Decrease in Child Welfare Numbers in 15 Years (25 September 2018). https://www.cbc.ca/news/canada/manitoba/manitoba-cfs-decrease-1.4837846.

[60] Canadian Press Child Apprehension Laws to be Amended so Kids can’t be Taken Because of Poverty (7 November 2018). https://globalnews.ca/news/4638801/child-apprehension-laws-to-be-amended-so-kids-cant-be-taken-because-of-poverty/.

[61] Gaetz S. (2010). The struggle to end homelessness in Canada: How we created the crisis, and how we can end it. Open Health Serv. Policy J..

[62] O’Grady B., Gaetz S., Buccieri K. (2011). Can I See Your ID? The Policing of Youth Homelessness in Toronto.

[63] O’Brien K., Colquhoun H., Levac D., Baxter L., Tricco A.C., Straus S., Wickerson L., Nayar A., Moher D., O’Malley L. (2016). Advancing scoping study methodology: A web-based survey and consultation of perceptions on terminology, definition and methodological steps. BMC Health Serv. Res..

[64] Pauly B. (2008). Harm reduction through a social justice lens. Int. J. Drug Policy.

